# AITC inhibits fibroblast-myofibroblast transition via TRPA1-independent MAPK and NRF2/HO-1 pathways and reverses corticosteroids insensitivity in human lung fibroblasts

**DOI:** 10.1186/s12931-021-01636-9

**Published:** 2021-02-12

**Authors:** Jennifer Maries Go Yap, Takashi Ueda, Yoshihiro Kanemitsu, Norihisa Takeda, Kensuke Fukumitsu, Satoshi Fukuda, Takehiro Uemura, Tomoko Tajiri, Hirotsugu Ohkubo, Ken Maeno, Yutaka Ito, Testsuya Oguri, Shinya Ugawa, Akio Niimi

**Affiliations:** 1 Department of Respiratory Medicine, Allergy and Clinical Immunology, Nagoya City University Graduate School of Medical Sciences, 1 Kawasumi, Mizuho-cho, Mizuho-ku, Nagoya, 467-8601 Japan; 2 Department of Anatomy and Neuroscience, Nagoya City University Graduate School of Medical Sciences, Aichi, Japan

**Keywords:** TRPA1, AITC, ERK1/2 MAPK pathway, NRF2/HO-1 pathway

## Abstract

**Background:**

Little is known on the role of transient receptor potential ankyrin 1 (TRPA1) in fibroblast—myofibroblast transition (FMT) that can lead to airway remodeling which is a major problem for severe asthma and fibrosis. Thus, this study investigated the effect of TRPA1 modulators on transforming growth factor beta 1(TGF-β1) -treated lung fibroblasts.

**Methods:**

MRC-5 cells were preincubated with TGF-β1 for 24 h. TRPA1 agonist or antagonist were added and further incubated for 24 h. The changes in TRPA1 and alpha-smooth muscle actin (α-SMA) expressions by stimuli were evaluated using qRT-PCR, western blot and immunohistochemical analyses. Statistical significance was determined by using one- or two-way ANOVA, followed by Bonferroni’s post hoc analysis for comparison of multiple groups and paired 2-tailed Student’s t-test between 2 groups.

**Results:**

MRC-5 cells treated by TGF-β1 significantly upregulated α-SMA mRNA expressions (*P* < 0.01), but downregulated TRPA1 gene expression (*P* < 0.001). Post-treatment of TRPA1 activator, allyl isothiocyanate (AITC), after TGF-β1 significantly downregulated the α-SMA gene induction (*P* < 0.01 at 24 h), protein expression (*P* < 0.05) and immunoreactivity with stress fibers (*P* < 0.05). On the other hand, TRPA1 antagonist HC-030031 did not prevent this effect, and instead tended to facilitate the suppressive effect of AITC when co-stimulated. AITC significantly increased phosphorylated- extracellular signal-regulated kinase (ERK) 1/2 and heme oxygenase (HO)-1 protein expressions (*P* < 0.05) in TGF-β1-treated cells. Combined inhibition with ERK1/2 mitogen-activated protein kinase (MAPK) and nuclear factor erythroid 2-related factor (NRF2) almost completely reversed AITC-induced α-SMA suppression (*P* < 0.05). Dexamethasone was not able to inhibit the upregulated α-SMA induction by TGF-β1. However, AITC improved dexamethasone-insensitive myodifferentiation in the presence of the corticosteroid (*P* < 0.01).

**Conclusion:**

We found that AITC exerts protective effect on TGF-β1-induced α-SMA induction by activating ERK1/2 MAPK and NRF2/HO-1 pathways in lung fibroblasts. It also overcomes corticosteroids insensitivity in TGF-β1-induced α-SMA induction. TRPA1 antagonist modulates the suppressive effect, but not prevent it. AITC and TRPA1 antagonist may be therapeutic agents in treating chronic respiratory diseases.

## Background

Chronic respiratory diseases such as asthma, chronic obstructive pulmonary disease (COPD), and idiopathic fibrosis (IPF) affect approximately 545 million in the world and account for 3.9 million deaths [1]. Although recommended management and treatment against these diseases were already stated in the guidelines [2–4], the unspecific and lack of treatments response among these patients [5, 6] suggest that there is a need for further understanding of the factors that underlie severity of these diseases.

Airway remodeling is strongly associated with the pathophysiology of asthma, COPD, and IPF [7–9]. Residing fibroblasts in the connective tissues of the bronchi are the most common source of myofibroblasts in which they transform their phenotype synthesizing extracellular matrix proteins and alpha-smooth muscle actin (α-SMA)(visible in cells as stress fibers) in a phenomenon known as fibroblasts-to-myofibroblasts transition (FMT). Increased formation of myofibroblasts is observed in subepithelial remodeling in chronic respiratory diseases [10–12].

Transforming growth factor-beta (TGF-β) is an important FMT-inducing mediator [13] which also functions in regulating cell growth, morphogenesis, cell differentiation and apoptosis [14]. It facilitates pro-inflammatory responses and tissue remodeling in airways [15]. TGF- β was also identified as the main cytokine that induces augmented matrix deposition mainly through fibroblast recruitment and transformation in the IPF lung [16]. TGF-β can also stimulate and increase reactive oxygen species (ROS) production that leads oxidant and antioxidant imbalance which in turn can activate latent TGF-β developing a profibrogenic cycle [17]. Moreover, TGF- β1 is associated with corticosteroids insensitivity in chronic inflammatory diseases including asthma [18].

Transient receptor potential ankyrin 1 (TRPA1) is a major TRP channel in human lung fibroblasts. We have demonstrated that tumor necrosis factor-α (TNF-α) can regulate airway inflammation by modulating TRPA1 expression on lung fibroblasts [19]. TRPA1 is activated by an antioxidant substance, allyl isothiocyanate (AITC), along with a variety of risk factors for respiratory diseases, such as cigarette smoke extract, bacterial endotoxins and painful cold. Activation of myofibroblast TRPA1 channel by corticosteroids and pirfenidone exerts its antifibrotic effects in intestinal fibrosis [20]. It is thus possible that TRPA1 is involved in development of FMT, oxidant and antioxidant imbalance, and corticosteroids sensitivity in lung fibroblasts during FMT. However, there are no studies exploring the involvement of TRPA1 in lung fibroblasts on airway remodeling and fibrosis. Thus, this study investigated the effect of TGF-β on the expression of TRPA1 in lung fibroblasts. We also determine the response of fibroblasts stimulated by TGF-β1 to TRPA1 agonist (AITC) and antagonist (HC-030031). We further explored the potential role of AITC in the pathogenesis of fibrosis that leads to airway remodeling.

## Materials and methods

### Cell culture and treatment of cells

MRC-5 and HF19 cells were provided by RIKEN BRC through the National Bio-Resource Project of the MEXT/AMED, Japan (RCB021 and RCB0210) and were cultured in RITC80-7 medium supplemented with 10%FCS, penicillin, and streptomycin in 37 °C with 5% CO_2_. Cells were seeded in 6-well plate for qRT-PCR and protein expressions and in 4-chamber slides for immunostainings using 1% FBS RITC80-7 medium. Time-point experiments were done at 24, 48, and 72 h after TGF-β1 treatment (5 ng/mL) and confirmed that 24 h treatment with TGF-β1 already upregulated gene expressions of α-SMA and collagen type 1 alpha 1 chain (Col1A1) (Additional file 1: Figure S1). Cells were initially incubated with TGF-β1 (5 ng/mL) for 24 h to induce FMT. The TGF-β1- treated cells were then stimulated with HC-030031(10 µM) or AITC (10 µM) and were incubated further for 24 h in the presence of TGF-β1. Cells were incubated for a total of 48 h before harvest.

### RNA Isolation and cDNA synthesis

Total RNA was isolated using ISOGEN reagent according to manufacturer's protocol (Fujifilm Wako, Osaka, Japan). cDNA was synthesized using the Superscript IV VILO Master Mix with ezDNase kit following the manufacturer's protocol (Invitrogen by Thermo Fisher Scientific, USA). Total RNA concentration of 1500 ng was used to synthesize cDNA.

### Quantitative RT-PCR of Target Genes

Quantitative RT-PCR of target genes were analyzed using 7900HT Fast real time PCR system (Applied biosystems Thermo Fisher Scientific, USA). We established specific primers as described previously (Additional file 1: Table S1) [19]. Relative gene expressions were calculated using 2^−ΔΔCt^ method after normalized with β-actin.

### Immunostaining

MRC-5 cells mounted on slides were air dried for 10 min and fixed in 4% paraformaldehyde for 15 min. Slides were then blocked with 5% normal donkey serum in phosphate buffered saline (PBS) containing 0.3% Triton X-100 for 30 min and were incubated overnight with α-SMA antibody (Table 1) at 4 ˚C. Cells were then washed with PBS and incubated with anti-mouse Alexa 488 secondary antibody for 1 hr. Immunostained cells were observed and analyzed under NIKON A1RS + confocal microscope (NIKON, Japan).

**Table 1:** List of antibodies used for western blot analysis

Primary and Secondary antibodies		Size (kDa)	Code
α-SMA	Monoclonal mouse anti-human smooth muscle actin, clone 1A4	43	M0851, DAKP/Agilent
GAPDH	D16H11 XP Rabbit mAb	37	5174, Cell Signaling Technology, MA, USA
Smad2/3	D7G7 XP Rabbit mAb	52, 60	8685, Cell Signaling Technology, MA, USA
Smad 2	D43B4 XP Rabbit mAb	60	5339, Cell Signaling Technology, MA, USA
Smad 3	C67H9 Rabbit mAb	52	9523, Cell Signaling Technology, MA, USA
Phospho-Smad 2 (Ser465/467)	138D4 Rabbit mAb	60	3108, Cell Signaling Technology, MA, USA
Phospho-Smad 3 (Ser423/425)	C25A9 Rabbit mAb	52	9520, Cell Signaling Technology, MA, USA
ERK1/2 (p44/42) MAPK	137F5 Rabbit mAb	46,54	4695, Cell Signaling Technology, MA, USA
p38 MAPK	D13E1 XP Rabbit mAb	40	8690, Cell Signaling Technology, MA, USA
SAPK/JNK	Rabbit Ab	46,54	9252, Cell Signaling Technology, MA, USA
Phospho-ERK1/2 (p44/42) MAPK (Thr202/Tyr204)	D13.14.4E XP Rabbit mAb	42, 44	4370, Cell Signaling Technology, MA, USA
Phospho-p38 MAPK (Thr180/Tyr182)	D3F9 Xp Rabbit mAb	40	4511, Cell Signaling Technology, MA, USA
Phospho-SAPK/JNK (Thr 183/Tyr 185)	81E11 Rabbit mAb	46,54	4668, Cell Signaling Technology, MA, USA
HO-1	E3F4S Rabbit mAb	28	43,966, Cell Signaling Technology, MA, USA

### Western blot analysis

Western blot analysis was performed as described previously [19], except for loaded protein volume (10 μg). Membranes were incubated with anti-GAPDH [4967S, Cell Signaling Technology (CST), Japan] or primary antibodies (Table 1) and followed by anti-rabbit IgG HRP-conjugated antibody (7074S, CST) and/or anti-mouse IgG HRP-conjugated antibody (W402B, Promega, USA). The bands were detected using chemiluminescence kit (ECL detection system; GE healthcare, USA) and were quantified using Image J software.

### Wound healing assay

MRC-5 cells were starved for 12 h in serum free medium and were grown to 80% confluence in 6-well plates. They were scratched by a 200 µL pipette tip to create gaps with uniform size. Cells were then washed twice with PBS to remove floating cells and were exposed in the presence of absence of 5 ng/mL TGF-β1. Cells were also treated with or without 10 µM AITC and/or 10 µM HC-030031 in serum free medium for 24 h. The number of migrating cells in the gap were counted in three fields from each treatment under a light microscope.

### Statistical analysis

Data were represented as means ± SEM and were analyzed using GraphPad Prism 5 statistical software. Statistical significance was determined by using one- or two-way ANOVA, followed by Bonferroni’s post hoc analysis for comparison of multiple groups and the paired 2-tailed Student’s t-test between 2 groups. *P* < 0.05 was considered statistically significant.

## Results

### TGF-β1 upregulated remodeling related genes expressions and downregulated TRPA1 gene expression

**Fig. 1:**
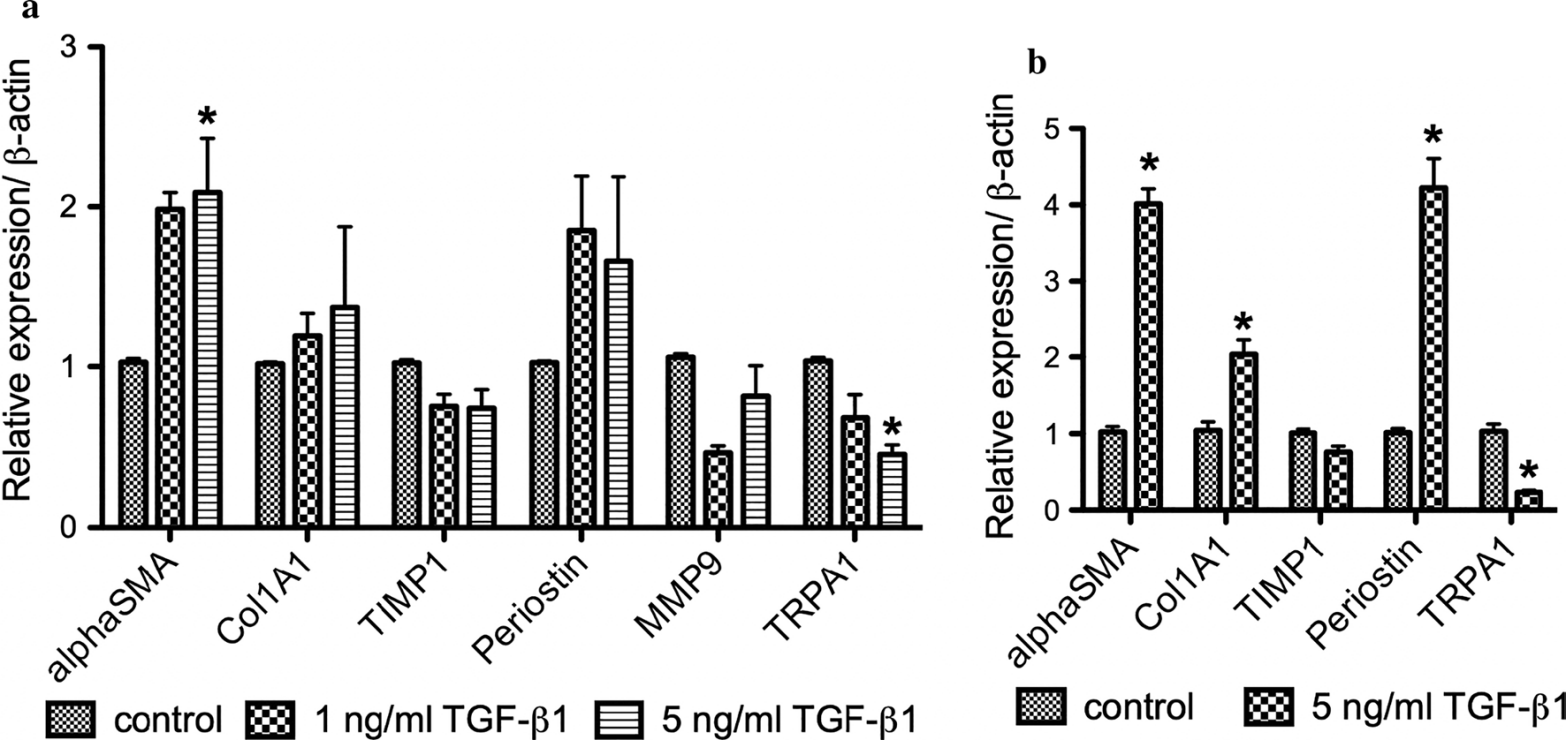
Relative gene expressions of TRP channels and remodeling gene markers after TGF-β1 stimulation in lung fibroblasts. MRC-5 cells (**a**) and HF19 cells (**b**) were treated with different concentrations (1 or 5 ng/mL) of TGF-β1. Gene expressions of TRP channels and remodeling gene markers were determined by qRT-PCR. β-actin was used to normalize the expressions. (*P < 0.05 compared with the respective control). N = 4

First, we investigated the effect of TGF-β1 on expressions of remodeling related genes such as α-SMA, Col1A1, tissue inhibitor matrix metalloproteinase 1 (TIMP1), periostin, and matrix metalloprotease 9 (MMP9) by using qRT-PCR. Treatment of MRC-5 cells with different concentrations of TGF-β1 (1 and 5 ng/mL) for 24 h significantly upregulated the gene expressions of α-SMA (*P* < 0.05), and was prone to increase gene expression of Col1A1 and periostin (Fig. 1a). We also confirmed the TGF-β1-induced FMT in HF19 another human lung fibroblast cell line (Fig. 1b). These support the previous finding that TGF-β1 promotes FMT in lung fibroblasts [7].

Recently, we reported that TRPA1 gene expression was upregulated upon treatment with TNF-α, another mediator for airway inflammation in chronic respiratory diseases [19]. Therefore, we also evaluated the effect of TGF-β1 on the gene expressions of TRPA1 in lung fibroblasts. Contrary to TNF-α treatment, MRC-5 cells treated with TGF-β1 significantly downregulated TRPA1 gene expressions (*P* < 0.05) (Fig. 1a). We also checked the gene expression in HF19 cells and obtained similar results (Fig. 1b). Unlike TNF-α, TGF-β1 could negatively regulate TRPA1 gene expression during the process of FMT in lung fibroblasts.

### AITC suppressed TGF-β1-induced α-SMA expression and cell migration in lung fibroblasts

**Fig. 2:**
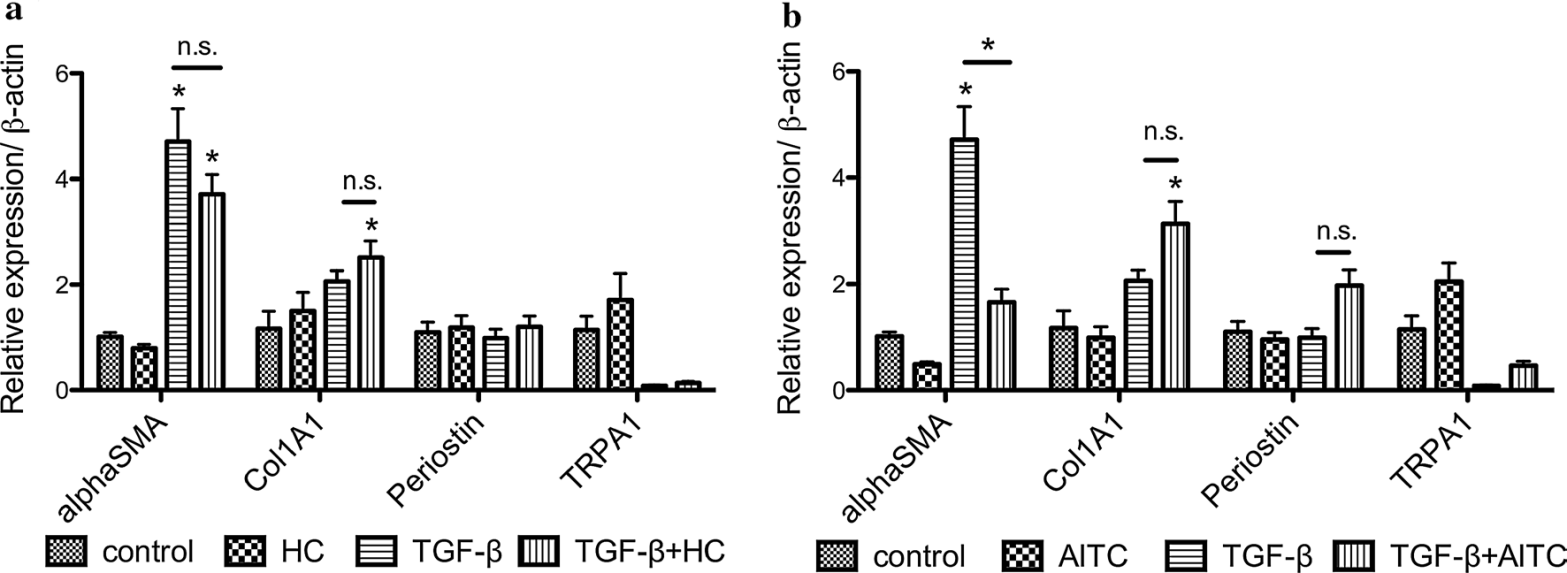
Effect of AITC and selective TRPA1 antagonist HC-030031 on remodeling gene markers after TGF-β1 stimulation. **a** MRC-5 cells were incubated with TGF-β1 (5 ng/mL) for 24 h and then treated with HC-030031 (30 μM) for 24 h (TGF-β + HC) in the presence of TGF-β1. TGF-β1 significantly increased α-SMA gene expression (*P < 0.05) and were not inhibited by HC-030031. N = 3. **b** MRC-5 cells were incubated with TGF-β1 (5 ng/mL) for 24 h and then treated with AITC (30 μM) for 24 h (TGF-β + AITC) in the presence of TGF-β1. The TGF-β1-induced α-SMA was significantly inhibited by AITC (*P < 0.01, * without bar means the results when compared with the respective control). N = 3

To examine the role of TRPA1 channel in FMT induced by TGF-β1 in lung fibroblasts, we used allyl isothiocyanate (AITC), a pungent chemical that is an ingredient of wasabi and mustard oil and is well known activator of the TRP channel, and HC-030031, a selective TRPA1 antagonist. MRC-5 cells were treated with AITC or HC-030031 for 24 h after TGF-β1 application. RNA samples were collected at 48 h after TGF-β1 for qRT-PCR analysis. We found that HC-030031 failed to reduce TGF-β1-induced α-SMA, collagen, and periostin gene expressions (Fig. 2a). In contrast, AITC significantly downregulated TGF-β1-induced α-SMA gene expression even in delayed application (*P* < 0.01 at 24 h) (Fig. 2b).

**Fig. 3:**
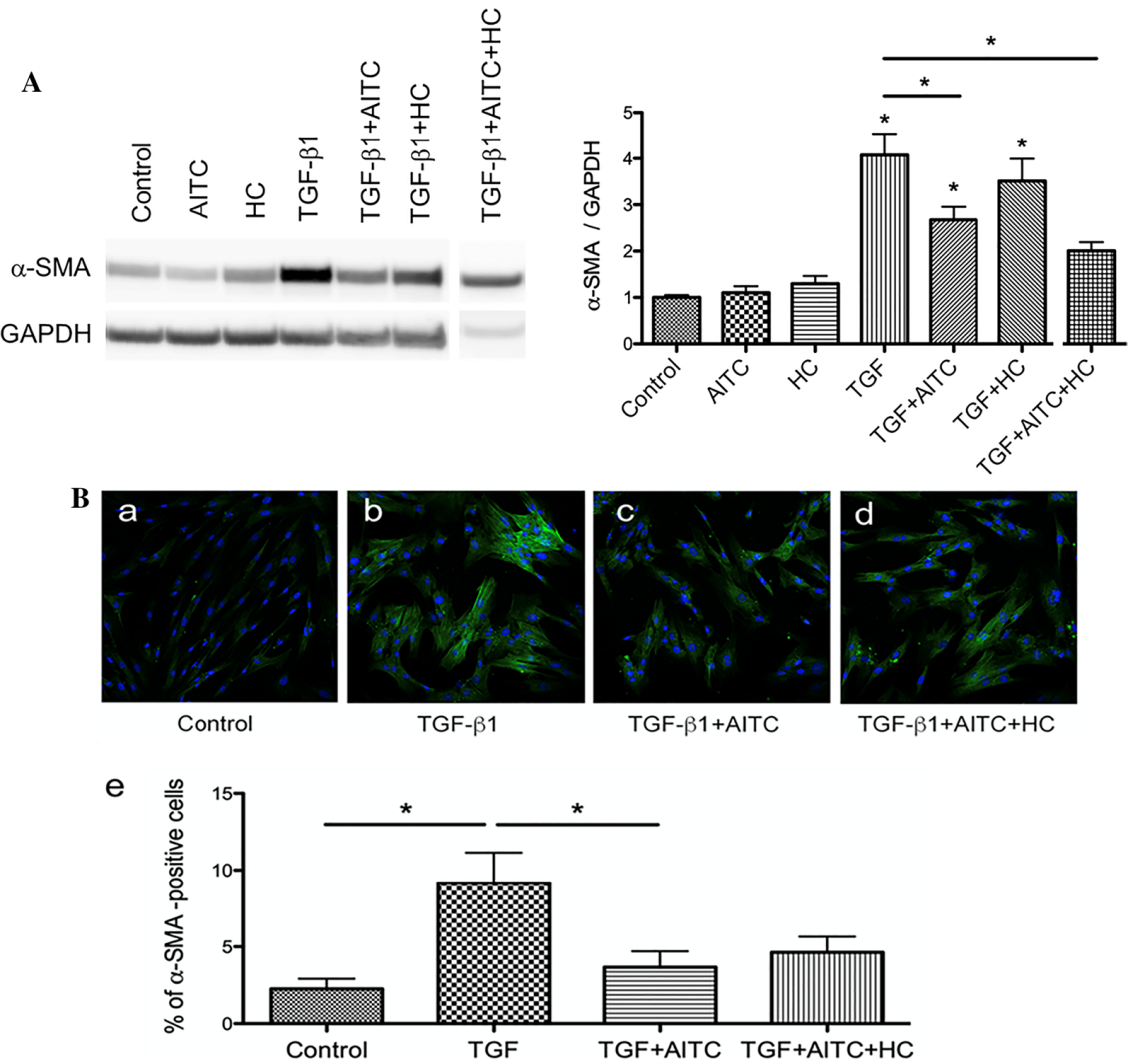
Effect of TRPA1 modulators on protein expression and immunoreactivity of α-SMA after TGF-β1 stimulation. **a** Western blot analysis of α-SMA protein expression after treatment with AITC (10 μM), HC-030031 (10 μM) or AITC (10 μM) + HC-030031 (10 μM) for 24 h in the latter with TGF-β1 incubation for 48 h where TGF-β1-induced α-SMA protein expression was decreased by AITC (*P < 0.05 at TGF vs TGF + AITC or TGF + AITC + HC). N = 5. **b** Immunohistochemical results showing α-SMA protein expression with stress fibers in cells after similar treatment to (**a**). (e) Summary of quantification of α-SMA positive cells. TGF-β1 increased α-SMA-immunoreactive cells and AITC significantly reduced number of the positive cells (*P < 0.05, * without bar; compared with control). N = 6

TGF-β1 induces α-SMA protein expression with stress fibers, a feature of myofibroblasts in process of FMT [21]. Treatment of MRC-5 cells with 5 ng/mL TGF-β1 for 48 h was sufficient to induce upregulation of α-SMA protein (Fig. 3a). Treatment with 10 µM AITC for 24 h after TGF-β1 significantly inhibited the upregulated α-SMA protein expression (*P* < 0.05). However, the suppressive effect of AITC was not reversed by simultaneous administration of 10 µM HC-030031. Instead, the TRPA1 antagonist tended to decrease the exacerbated α-SMA protein expression when stimulated together with AITC (Fig. 3a). We also observed immunohistochemical changes of α-SMA proteins after TGF-β1 in the presence or absence of AITC under a confocal laser scanning microscope (Fig. 3b). TGF-β1 significantly increased α-SMA-positive cells with stress fibers (Fig. 3b; b and e, *P* < 0.01), which were suppressed prominently by co-treatment with AITC (Fig. 3b; c and e, *P* < 0.05). However, HC-030031 failed to counteract the suppressive effect of AITC (Fig. 3b, d and e).

**Fig. 4:**
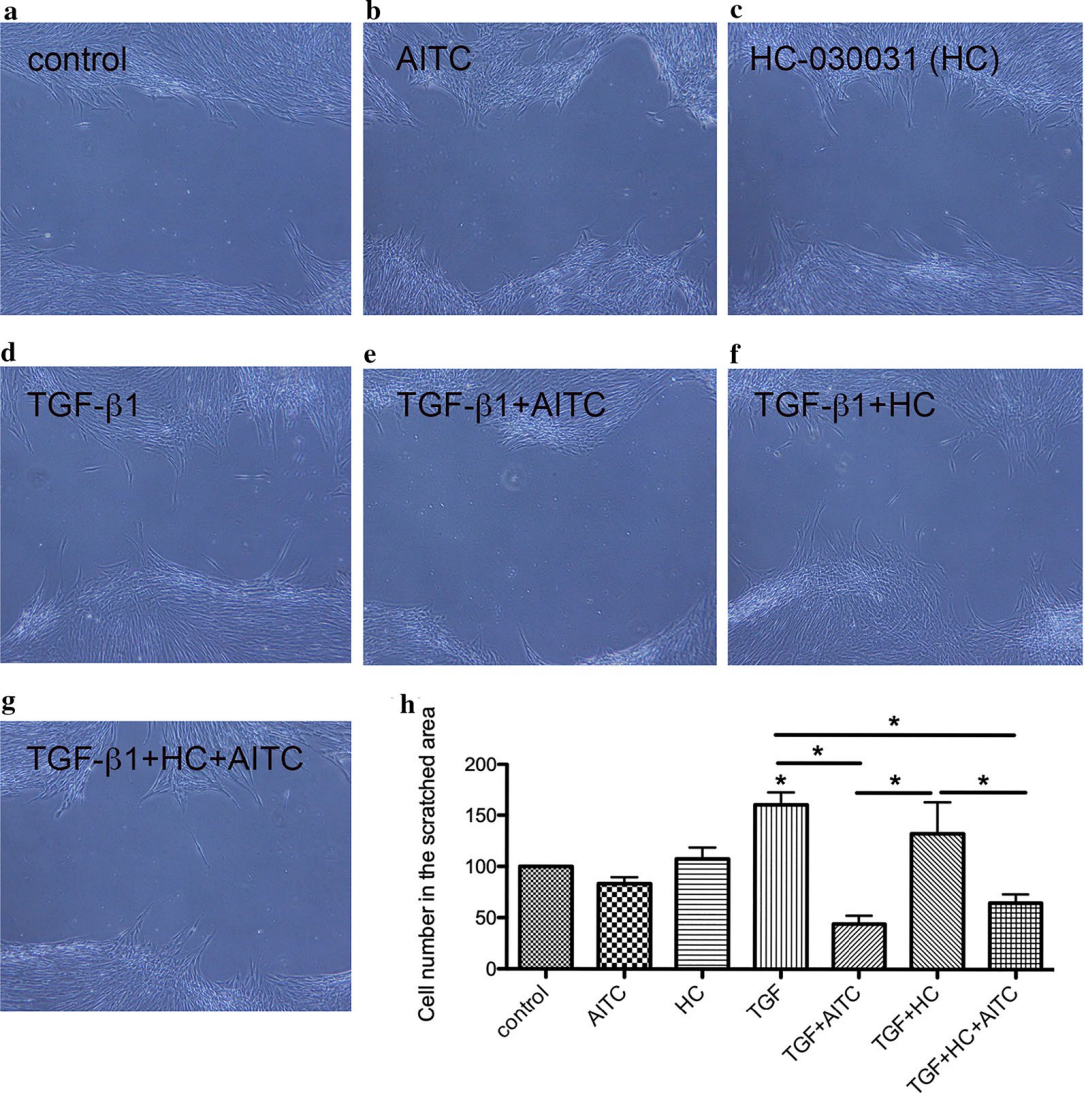
Effect of AITC and selective TRPA1 antagonist HC-030031 on cell migration after TGF-β1 stimulation. **a**–**g** Representative images under light microscope and **h** quantification of cell number in the gap area. Cells were manually scratched with a pipette tip, incubated with **b** AITC alone or **c** HC-030031 alone, and pretreated with **d** TGF-β1 (5 ng/ml) and incubated with **e** AITC (10 μM) or **f** HC-030031 (10 μM) or **g** AITC (10 μM) + HC-030031 (10 μM) for 24 h. TGF-β1 promoted migration of MRC-5 cells and AITC significantly inhibited the migration of TGF-β1-treated MRC-5 cells. HC-030031 did not hinder the migration of cells and was not able to reverse the suppressive effect of AITC (*P < 0.05). N = 7

We also examined the effect of AITC on cell migration because TGF-β1 is also known to activate cell migration [22] (Fig. 4). There was no significant difference between control (Fig. 4a) and AITC (Fig. 4b) or HC-030031 alone (Fig. 4c). As compared to control, TGF-β1 activated cell migration (Fig. 4d and h). AITC significantly inhibited the migration of TGF-β1-treated MRC-5 cells (*P* < 0.001) (Fig. 4e and h). Exposure of HC-030031 to TGF-β1-treated cells did not hinder the migration of cells (Fig. 4f) and was not able to reverse the suppressive effect of AITC (Fig. 4g).

We performed calcium imaging analysis whether AITC could induce calcium response in lung fibroblasts after treatment of TGF-β1 for 24 h and HC-030031 could inhibit it. AITC exhibited significant calcium influx with EC_50_ of 2.7–3.6 μM comparable to untreated in the fibroblasts treated with TGF-β1. Moreover, the calcium response was significantly inhibited by HC-030031, a selective TRPA1 antagonist (Additional file 1: Figure S2). Signifying the presence of TRPA1 independent pathway to inhibit TGF-β1-induced FMT by AITC.

### AITC activated ERK MAPK pathway independent of Smad2/3 pathway during TGF-β1-induced lung FMT

**Fig. 5:**
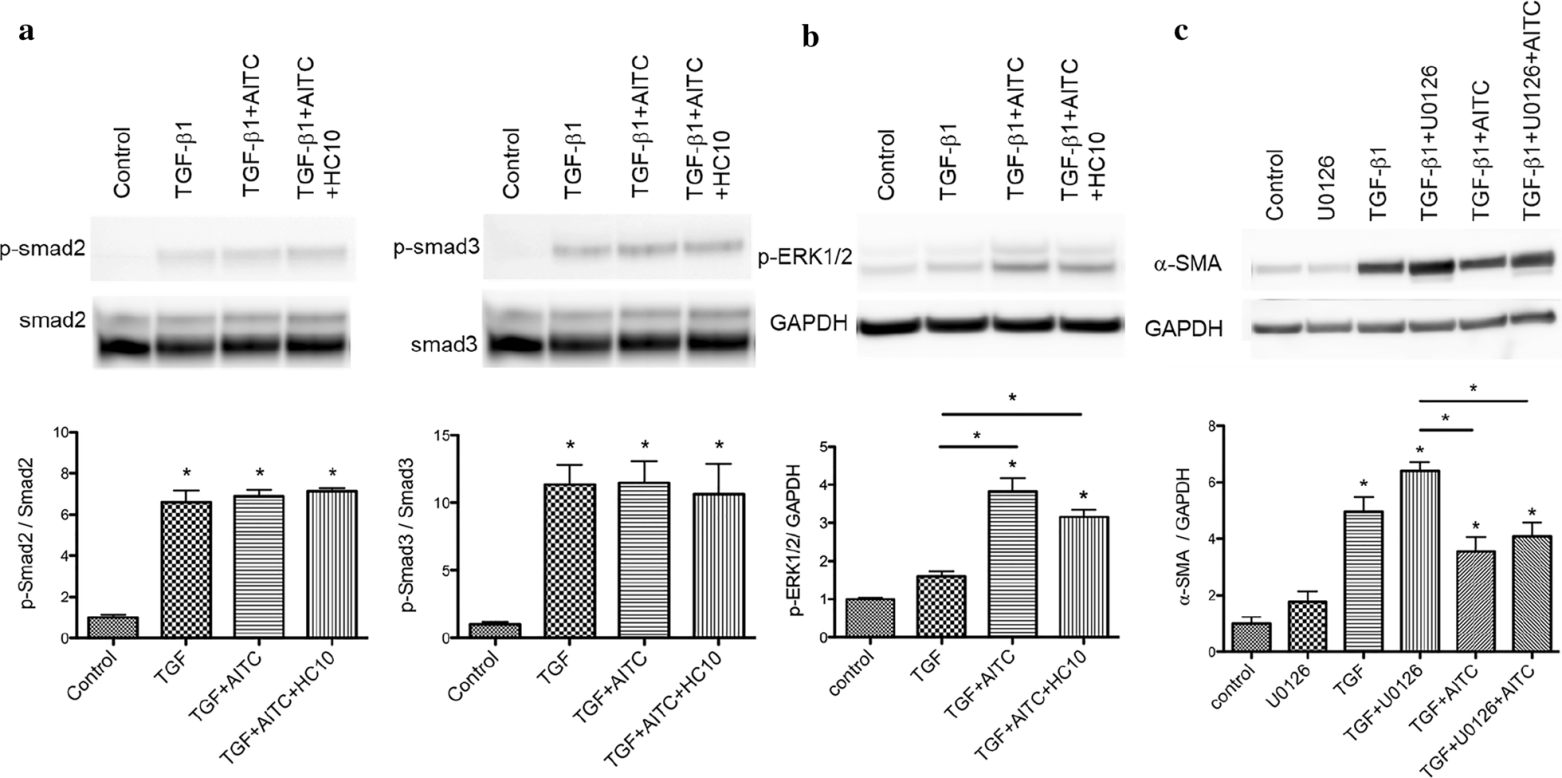
Phosphorylation of Smad2/3 and MAPK pathways and inhibition of ERK1/2 in TGF-β1-treated fibroblasts after AITC and HC-030031 stimulation. Western blot analysis of **a** phosphorylated Smad 2 (p-Smad2) and Smad 3 (p-Smad3) proteins 1 h and **b** phosphorylated ERK1/2 MAPK (p-ERK1/2) 24hrs after treatment with AITC (10 μM) or AITC (10 μM) + HC-030031 (10 μM) during TGF-β1 stimulation. **c** Western blot analysis of α-SMA protein expression after treatment with U0126, ERK1/2 inhibitor (10 μM), AITC (10 μM), or AITC (10 μM) + U0126 (10 μM) during TGF-β1 stimulation. (*P < 0.05, * without bar; compared with the respective control). N = 6

Smad2/3 phosphorylation could be prominent in early stage after TGF-β1 stimulation [23]. Therefore, we determined if the suppressive effect of AITC may be regulated by Smad 2 or Smad 3 pathway. We examined the effect of AITC on Smad2/3 phosphorylation in MRC-5 fibroblasts incubated with AITC for 1 hr after preincubation with TGF-β1. TGF-β1 apparently phosphorylated Smad 2 and Smad 3 as seen in the upregulation (*P* < 0.001), but AITC or HC-030031 did not affect Smad2/3 phosphorylation (Fig. 5a).

We next checked for non-Smad signaling pathways of TGF-β1. Mitogen-activated protein kinase (MAPK) signaling pathways have also been associated with development or inhibition of fibrotic responses through propagating signals initiated by growth factors such as TGF-β1 [24, 25]. Delayed application of AITC 24 h after TGF-β1 notably induced phosphorylation of ERK1/2 in MRC-5 cells treated with TGF-β1 at 48 h (Fig. 5b). Treatment with HC-030031 did not affect ERK1/2 phosphorylation. In contrast, no phosphorylated JNK and p38 were detected in the present study (data not shown). To check the possible contribution of ERK1/2 in the suppression of TGF-β1-induced α-SMA by AITC, we utilized U0126, an ERK inhibitor. The inhibitor slightly upregulated TGF-β1-induced α-SMA, supporting the finding that ERK inhibition further promoted FMT [24, 25]. AITC significantly reversed the aggravated effect of ERK inhibition on TGF-β1 induced α-SMA expression (Fig. 5c). Collectively, ERK1/2 MAPK pathway, but not Smad pathway, could contribute to the suppression of TGF-β1-induced FMT by AITC.

### AITC remarkably increased HO-1 protein expression through activation of ERK MAPK pathway

**Fig. 6:**
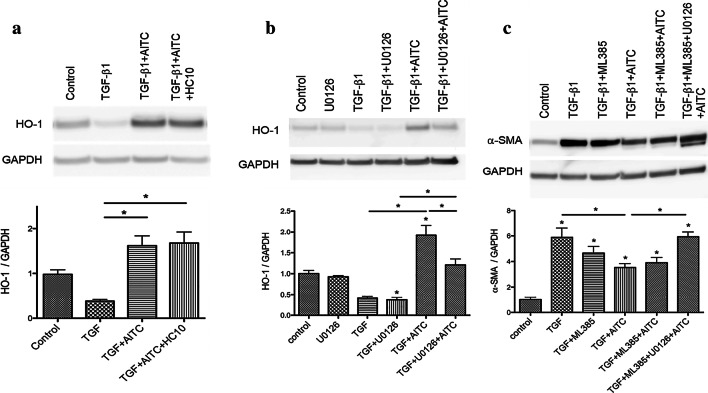
Effect of AITC, HC-030031, ERK1/2 and NRF2 inhibition on HO-1 and α-SMA expressions after TGF-β1 stimulation. Western blot analysis of HO-1 protein expression after treatment with **a** AITC (10 μM) or AITC (10 μM) + HC-030031 (10 μM), (B) U0126, ERK1/2 inhibitor (10 μM) for 24 h during TGF-β1 stimulation. **c** Western blot analysis of α-SMA protein expression after treatment with U0126 and/or ML385, NRF2 inhibitor (10 μM) for 24 h during TGF-β1 stimulation. (*P < 0.05, * without bar; compared with the respective control). N = 3

Previous studies have demonstrated the protective role of heme oxygenase-1 (HO-1) on tissue remodeling [26–29]. We next determined the protein expression levels of HO-1 in TGF-β1-treated MRC-5 cells incubated with AITC or HC-030031. TGF-β1 triggered oxidant and antioxidant imbalance by inhibiting HO-1 protein expression (Fig. 6a). Exposure with AITC significantly enhanced protein expressions of HO-1 in TGF-β1-treated MRC-5 cells, whereas HC-030031 with AITC did not have any changes (Fig. 6a). Increased expression of HO-1 by AITC was inhibited by ERK inhibitor, U0126 (Fig. 6b). Thus, AITC could suppress TGF-β1-induced FMT by activating HO-1 and ERK1/2 MAPK pathways.

### AITC may activate antioxidant Nrf2/HO-1 pathway

We further examined the involvement of NRF2 signaling using NRF2 inhibitor, ML-385, since it is well known to modulate HO-1 expression. TGF-β1-treated MRC-5 cells were incubated with ML-385 and/ or AITC for 24 h before harvesting protein for western blot analysis. Incubation with ML-385 showed no apparent reversal effect on AITC-induced α-SMA suppression in TGF-β1-treated cells (Fig. 6c), though it reduced HO-1 protein expression (data not shown). There is no significant difference between TGF + AITC and TGF + ML385 + AITC, although NRF2 inhibition tended to partially cancel AITC- induced α-SMA inhibition (Fig. 6c). Finally, application of both NRF2 and ERK inhibitors significantly reversed AITC-induced protective effect to TGF-β1-induced α-SMA induction (Fig. 6c). AITC could activate both ERK1/2 MAPK and NRF2/HO-1 pathways in suppressing TGF- β1-induced expression of α-SMA.

### AITC significantly improved dexamethasone insensitivity

**Fig. 7:**
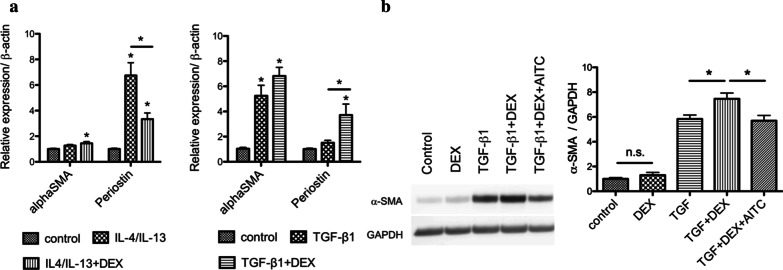
Effect of AITC on glucocorticoid sensitivity after IL-4/IL-13 or TGF-β1 treatment. Relative gene expressions of α-SMA and periostin demonstrating **a** the sensitivity after treatment with dexamethasone (DEX) (10 μM) for 24 h in the second half during IL-4/IL-13 (20 ng/mL each) stimulation for 48 h and **b** the sensitivity to dexamethasone (DEX) (10 μM) for 24 h in the second half during TGF-β1 (5 ng/mL) stimulation for 48 h (*P < 0.05, * without bar; compared with the respective control). N = 3. **c** Western blot analysis of α-SMA protein expression where AITC (10 μM) significantly reduced TGF-β1 (5 ng/mL)-induced and DEX (10 μM)-insensitive α-SMA protein expression (*P < 0.01). N = 5

Generally, TGF-β1 impairs corticosteroid action. Corticosteroids were not effective in modulating fibrotic processes that leads to remodeling [23, 30–32]. To confirm this phenomenon, we treated MRC-5 cells with either TGF-β1 or IL-4/IL-13 and incubated with dexamethasone. Figure 7a showed that IL-4/IL-13 treatment increased periostin mRNA levels (*P* < 0.0001) and the upregulated expression was significantly blocked by dexamethasone (*P* < 0.01). In contrast, TGF-β1-induced gene expressions of α-SMA and periostin were not blocked and was further exaggerated by dexamethasone (Fig. 7a), reflecting refractoriness to corticosteroids against TGF-β1-induced airway inflammation. Upon confirming refractoriness to corticosteroids, we determined the effect of AITC on this phenomenon. Indeed, AITC significantly inhibited TGF-β1- and dexamethasone- induced protein expression of α-SMA (*P* < 0.01) (Fig. 7b), suggesting that AITC recovers response to corticosteroids against TGF-β1-induced airway inflammation.

## Discussion

The present study demonstrated a possible role of TRPA1 and its modulators on FMT, an indicator of airway remodeling in human lung fibroblasts. First, we showed for the first time the anti-remodeling effect of TRPA1 activator AITC in lung fibroblast by suppressing TGF-β1-induced α-SMA gene and protein expressions as well as impeding cell migration and reducing stress fiber formation. Second, this suppressive effect was mediated through ERK1/2 MAPK and NRF2/HO-1 antioxidant pathway. Third, TRPA1 antagonist do not hinder the effect of AITC instead tends to facilitate it. Finally, AITC also has the capacity to inhibit the upregulated α-SMA expression resistant to dexamethasone in the presence of corticosteroids. The possible application of both TRPA1 modulators may be applicable for alleviating airway remodeling and airway hypersensitivity in chronic respiratory diseases.

Our study indicated that AITC suppresses TGF-β1-induced α-SMA expression in TRPA1-independet mechanism. This result was in congruent with the recent finding that blockade of TRPA1 by A967079, another selective TRPA1 inhibitor, did not reduce myofibroblast contraction, changes in α-SMA stress fibers, nor FBS-induced wound healing [33, 34]. In contrast, our calcium imaging analysis confirmed that AITC actually induced calcium response in lung fibroblasts treated with TGF-β1 for 24 h and HC-030031 significantly blocked it. Note that HC-030031 tended to downregulate TGF-β1-induced α-SMA protein expression when co-treated with AITC (Fig. 3a). These suggest that TRPA1 channel is still functional and may have a role during early phase of FMT. Myofibroblasts express abundant inflammatory mediators, cytokines and growth factors such as TGF-β, granulocyte–macrophage colony-stimulating factor (GM-CSF), interleukins (IL-1, IL-6, IL-8) and vascular endothelial growth factor (VEGF) [7]. We previously reported that TRPA1 channel was involved in excessive IL-8 release in TNF-α-stimulated MRC-5 cells [19]. We should examine the role of TRPA1 on cytokine release during FMT in future study. Thus, there could be more mechanisms present that are either TRPA1-dependent or independent which synergistically contribute to the phenomenon found in lung fibroblasts during FMT. Further studies are needed to elucidate this effect of TRPA1 antagonist on FMT.

Previous studies reported that AITC acted through TRPA1 [35–37], but there are some reports describing TRPA1-independent AITC effect as well [38–40]. We determined that AITC could suppress FMT induced by TGF-β1 through the activation of ERK1/2 MAPK and HO-1/NRF pathways in TRPA1-independent manner. The suppressive effect of AITC was insensitive to HC-030031, whereas it was reversed by co-treatment with ERK1/2 inhibitor, U0126 and NRF2 inhibitor, ML385. These findings are in line with the previous reports, in which AITC enhanced phosphorylation of ERK and induced antioxidant enzymes such as NAD(P)H dehydrogenase [quinone] 1 (NOQ1), HO-1, NRF2 and γ-glutamylcysteine synthetase (γGCS) in NIH3T3 mouse dermal fibroblasts [41] and cigarette smoke extract (CSE)-exposed 16HBE human bronchial epithelial cells [42]. Activating NRF2 attenuated both OVA-induced allergic airway inflammation and bleomycin-induced pulmonary fibrosis in rodents [43, 44]. The present finding that AITC targets the NRF2/HO-1 pathway to reduce FMT in human lung fibroblasts could be of value as potential therapeutic strategy for treatment of chronic respiratory disease.

As previously reported, patients with severe asthma are less responsive to corticosteroids than those with mild asthma and consequently considered that corticosteroids resistance might be a mechanism contributing to asthma severity [45, 46]. As well as patients suffering from IPF and COPD were also initially prescribed with corticosteroids and showed no response to corticosteroid therapy [47, 48]. We also found that AITC was able to reverse the action of dexamethasone, which failed to inhibit TGF-β1-induced α-SMA proteins in lung fibroblasts (Fig. 7b). Recent studies demonstrated that the isothiocyanate sulforaphane (SFN) reversed corticosteroid resistance by modulating NRF2 pathway in cigarette smoke-exposed asthmatic mice [49] and in a mixed granulocyte mouse model of asthma [50]. AITC is known to be a structurally related compound of SFN and has a similar effect in activating NRF2/HO-1 pathway [41, 42]. Thus, it may be possible that AITC treatment could restore corticosteroids sensitivity by activating NRF2/HO-1 pathway in human lung fibroblasts. However, additional investigations are needed, since previous studies utilized different cells from lung fibroblasts and mouse models, in which AITC equally acts on a variety of non-specific cells such as epithelial and immune cells present.

This study has several limitations. First, mechanisms involving TRPA1 dependent and independent remains to be elucidated as this study was done in a single cell line only. We would like to further understand these mechanisms utilizing samples from patients suffering from chronic respiratory diseases. Second, the experimental conditions were limited to certain concentrations and time exposure which can only mimic certain level of fibrosis in cells. Third, α-SMA was the only marker investigated as it was the most reactive among tested. Further experimentations including other markers of airway remodeling and cytokine release would be needed to further verify the role of TRPA1 in human lung fibroblasts. Fourth, we should determine the molecular mechanisms involved in restoring corticosteroids sensitivity of AITC, as several molecular mechanisms that might be responsible for reduced corticosteroids [51, 52]. Fifth, utilizing experimental animal models are recommended for verification of the anti-remodeling effect of AITC.

## Conclusion

The present study reported for the first time that AITC suppressed TGF-β1 induced gene and protein expression of α-SMA through induction of HO-1 via ERK1/2 MAPK and NRF2 pathways. AITC also inhibited TGF-β1-induced and dexamethasone-insensitive α-SMA expression and may reverse corticosteroids resistance in lung fibroblasts. These results would be of great help to elucidate the mechanisms of protective role of AITC on FMT. AITC may be a prospect agent for airway remodeling which are hallmarks of chronic respiratory diseases such as asthma, COPD, and IPF.

## Supplementary Information

**Additional file 1: Figure S1.** Effect of TGF-β1 stimulation after different time points on α-SMA and Col1A1. Cells were stimulated with TGF-β1 (5 ng/mL) and after different endpoints (24, 48, 72 h) RNA samples were isolated for qPCR analysis. Relative gene expressions of α-SMA and Col1A1 were already upregulated after 24 h as well as after 48 and 72 h (*P < 0.05). N = 4. **Figure S2.** Effect of AITC and TRPA1 antagonist HC-030031 on calcium response after TGF-β1 stimulation. Calcium imaging analysis was performed in TGF-β1-treated human lung fibroblasts. AITC exhibited significant calcium influx with EC_50_ of 2.7 – 3.6 μM comparable to untreated in the fibroblasts treated with TGF-β1. HC-030031 significantly inhibited calcium response (*P < 0.05). **Table S1.** List of primers used for qPCR.

## Data Availability

The datasets used and/or analyzed during the current study are available from the corresponding author on reasonable request.

## Abbreviations

TRPA1: Transient receptor potential ankyrin 1
AITC: Allyl isothiocyanate
ERK: Extracellular signal-regulated kinase
MAPK: Mitogen-activated protein kinase
NRF2: Nuclear factor erythroid 2-related factor 2
HO-1: Heme oxygenase-1
α-SMA: Alpha-smooth muscle actin
Col1A1: Collagen type 1 alpha 1 chain
TIMP1: Tissue inhibitor matrix metalloproteinase 1
MMP9: Matrix metalloprotease 9
TGF-β: Transforming growth factor beta
TNF-α: Tumor necrosis factor-α
FMT: Fibroblasts-to-myofibroblasts transition
