# Supplementary material for: AITC inhibits fibroblast-myofibroblast transition via TRPA1-independent MAPK and NRF2/HO-1 pathways and reverses corticosteroids insensitivity in human lung fibroblasts
Source: Respir Res. 2021 Feb 12;22:51. doi: 10.1186/s12931-021-01636-9 (PMC7881560; doi:10.1186/s12931-021-01636-9)
Supplement: Supplementary file 1 — Additional file 1: Figure S1. Effect of TGF-β1 stimulation after different time points on α-SMA and Col1A1. Cells were stimulated with TGF-β1 (5 ng/mL) and after different endpoints (24, 48, 72 h) RNA samples were isolated for qPCR analysis. Relative gene expressions of α-SMA and Col1A1 were already upregulated after 24 h as well as after 48 and 72 h (*P < 0.05). N = 4. Figure S2. Effect of AITC and TRPA1 antagonist HC-030031 on calcium response after TGF-β1 stimulation. Calcium imaging analysis was performed in TGF-β1-treated human lung fibroblasts. AITC exhibited significant calcium influx with EC50 of 2.7 – 3.6 μM comparable to untreated in the fibroblasts treated with TGF-β1. HC-030031 significantly inhibited calcium response (*P < 0.05). Table S1. List of primers used for qPCR. [file 12931_2021_1636_MOESM1_ESM.docx]

**Additional file**

**AITC inhibits fibroblast-myofibroblast transition via TRPA1-independent MAPK and NRF2/HO-1 pathways and reverses corticosteroids insensitivity in human lung fibroblasts**

Jennifer Maries Go Yap1*, Takashi Ueda2*, Yoshihiro Kanemitsu1**, Norihisa Takeda1, Kensuke Fukumitsu1, Satoshi Fukuda1, Takehiro Uemura1, Tomoko Tajiri1, Hirotsugu Ohkubo1, Ken Maeno1, Yutaka Ito1, Testsuya Oguri1, Shinya Ugawa2 and Akio Niimi1

1Department of Respiratory Medicine, Allergy and Clinical Immunology, Nagoya City University Graduate School of Medical Sciences, Aichi, Japan

2Department of Anatomy and Neuroscience, Nagoya City University Graduate School of Medical Sciences, Aichi, Japan

* equally contributed to this work

**Corresponding author

**Figure S1. Effect of TGF-β1 stimulation after different time points on** **α-SMA and Col1A1**

Cells were stimulated with TGF-β1 (5 ng/mL) and after different endpoints (24, 48, 72 hrs) RNA samples were isolated for qPCR analysis. Relative gene expressions of α-SMA and Col1A1 were already upregulated after 24 hrs as well as after 48 and 72 hrs (**P* < 0.05). N = 4.

**Figure S2. Effect of AITC and TRPA1 antagonist HC-030031 on calcium response after TGF-β1 stimulation**

Calcium imaging analysis was performed in TGF-β1-treated human lung fibroblasts. AITC exhibited significant calcium influx with EC_50_ of 2.7 – 3.6 μM comparable to untreated in the fibroblasts treated with TGF-β1. HC-030031 significantly inhibited calcium response (**P* < 0.05).


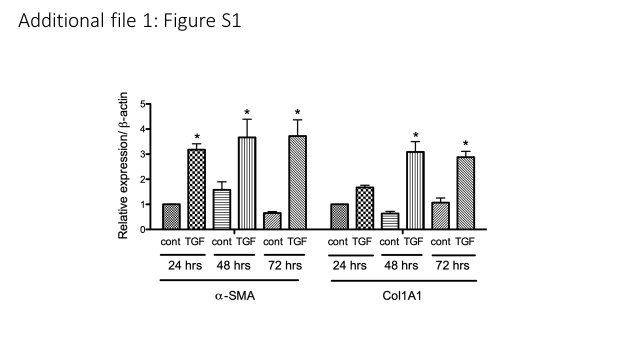


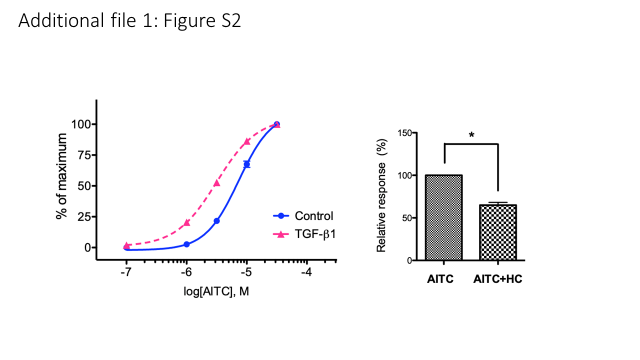


Additional file 1: Table S1. List of primers used for qPCR.

| Gene (GenBank#) | Forward sequence | Reverse sequence | bp size |
| --- | --- | --- | --- |
| Homo sapiens TRPA1: NM_007332.2 | CTCCTCTCCACATAGCTGTGCA  573-594 | GTGCACGCAATGATCACAGCTGT 674-696 | 124 |
| Homo sapiens COL1A1: NM_000088.3 | AGCTTTGTGGACCTCCGGCT  133-152 | GCAGGTGATTGGTGGGATGTCT  225-246 | 114 |
| Homo sapiens Periostin: NM_001135934.1 | TACTGGAAACCATCGGAGGCA  1461-1481 | CGGAATATGTGAATCGCACCGT 1563-1584 | 124 |
| Homo sapiens MMP9: NM_004994.2 | CTTTGGACACGCACGACGTCT  1992-2012 | TCAGGGCACTGCAGGATGTCAT 2115-2136 | 145 |
| Homo sapiens TIMP1: NM_003254.2 | GCTTCACCAAGACCTACACTGT  605-626 | CTGGAAGCCCTTTTCAGAGCCT  720-741 | 137 |
| Homo sapiens  α-SMA: NM_001613.3 | CATTGCCGACCGAATGCAGAA 1100-1120 | CCACCGATCCAGACAGAGTAT  1184-1204 | 105 |
| Homo sapiens  β-actin: NM_001101.3 | TGGCACCCAGCACAATGAAGATCA 1043-1066 | CTGCTTGCTGATCCACATCTGCT 1142-1164 | 122 |
